# How to make an eye clinic more accessible for people with low vision

**Published:** 2012

**Authors:** Jaya Srivastava

**Affiliations:** Low Vision Consultant, Spectrum Eye Care, Prasad Chambers, 169, Peters Road, Gopalapuram, Chennai, Tamil Nadu, India 600086.

**Figure:**
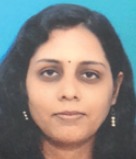
Jaya Srivastava

## Good patient flow

- The unit should be laid out in such a way that it is easy for patients to go from one part of the unit to another
- Some hospitals paint coloured lines on the floor which patients can follow. For example, a brightly coloured line may lead straight from registration to the first waiting area
- Remove obstacles that people with low vision may fall over or collide with.

## Use of colour, contrast, and lighting

- Use large, clear letters for all the signs in the department. Ensure there is good overall illumination and avoid creating glare, which could be caused by using shiny white tiles on the floor and walls. For signs, use colours with high contrast, e.g., white or yellow lettering on a black or dark background Before you make any changes, make sure people with low vision can read the signs!
- In the waiting area, use brightly coloured chairs, or paint them in a contrasting colour compared to the walls and floor. This will help people with low vision to find them and see the ones that are empty
- Use tape or paint to apply a thick line to the edges of steps to make them more visible. Use ramps with a handrail instead of stairs, if possible
- White hand basins and toilets against white tiles can make bathrooms very difficult to use. Change the colour of the walls and/or floor to improve contrast
- If there are lifts, put a brightly coloured arrow or ring around the call button, or paint the door a different colour (Figure 2).

**Figure 1:**
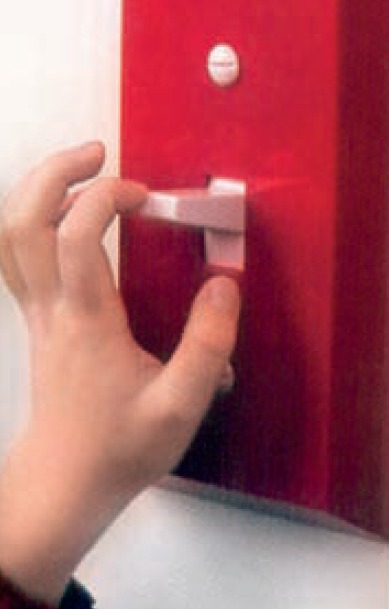


**Figure 2:**
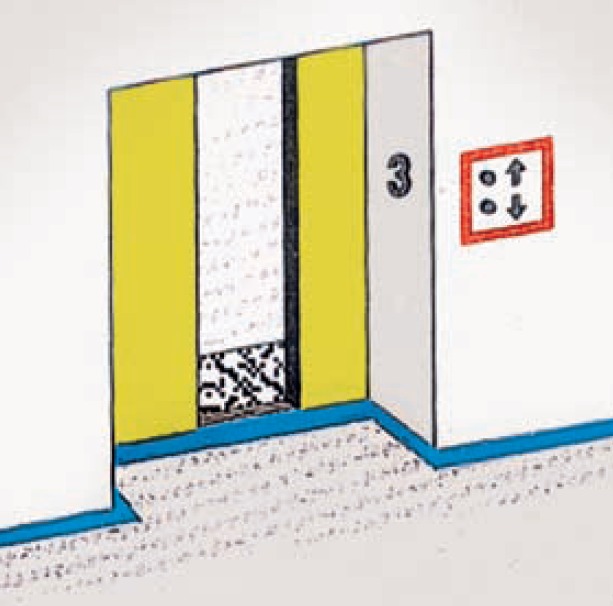


## Staff assisting someone with low vision

- Be patient: people with low vision may have visited many eye units or professionals already, and have told their stories many times before
- Be kind: people may initially be angry when they are told they have untreatable visual loss. Listen and be supportive, but do not give false hope.

